# Incidence and underreporting of osseous wrist and hand injuries on whole-body computed tomographies at a level 1 trauma center

**DOI:** 10.1186/s12891-021-04754-z

**Published:** 2021-10-11

**Authors:** L. Schmehl, A. Hönning, A. Asmus, S. Kim, S. Mutze, A. Eisenschenk, L. Goelz

**Affiliations:** 1 Department of Radiology and Neuroradiology, BG Klinikum Unfallkrankenhaus Berlin, Warener Str. 7, 12683 Berlin, Germany; 2 Center for Clinical Research, BG Klinikum Unfallkrankenhaus Berlin, Berlin, Germany; 3 Department of Hand-, Replantation- and Microsurgery, BG Klinikum Unfallkrankenhaus Berlin, Berlin, Germany; 4 Department of Hand Surgery and Microsurgery, University Medicine Greifswald, Greifswald, Germany; 5 Institute for Diagnostic Radiology and Neuroradiology, University Medicine Greifswald, Greifswald, Germany

**Keywords:** Osseous injuries, Wrist, Hand, Missed injuries, Underreporting, Whole-body computed tomographies, WBCT, Polytrauma

## Abstract

**Background:**

To investigate the incidence of osseous wrist and hand injuries on whole-body computed tomographies (WBCT) at an urban maximum-care trauma center, to report the number of missed cases in primary radiology reports, and to develop an algorithm for improved detection of these injuries.

**Methods:**

Retrospective analysis reviewing all WBCT for a period of 8 months for osseous wrist and hand injuries. (1) Reconstruction of hands/wrists in three planes (thickness 1–2 mm) and analysis by a blinded musculoskeletal radiologist. (2) Scanning of primary radiology reports and comparison to the re-evaluation. (3) Calculation of the diagnostic accuracy of WBCT during primary reporting. (4) Search for factors potentially influencing the incidence (trauma mechanism, associated injuries, Glasgow Coma Scale, artifacts). (5) Development of an algorithm to improve the detection rate.

**Results:**

Five hundred six WBCT were included between 01/2020 and 08/2020. 59 (11.7%) WBCT showed 92 osseous wrist or hand injuries. Distal intra-articular radius fractures occurred most frequently (*n* = 24, 26.1%); 22 patients (37.3%) showed multiple injuries. The sensitivity of WBCT in the detection of wrist and hand fractures during primary evaluation was low with 4 positive cases identified correctly (6.8%; 95% CI 1.9 to 16.5), while the specificity was 100% (95% CI 99.2 to 100.0). Forty-three cases (72.9%) were detected on additional imaging after clinical reassessment. Twelve injuries remained undetected (20.3%). Motorcycle accidents were more common in positive cases (22.0% vs. 10.1%, *p* = 0.006). 98% of positive cases showed additional fractures of the upper and/or lower extremities, whereas 37% of the patients without osseous wrist and hand injuries suffered such fractures (*p* < 0.001). The remaining investigated factors did not seem to influence the occurrence.

**Conclusion:**

Osseous wrist and hand injuries are present in 11.7% on WBCT after polytrauma. 93.2% of injuries were missed primarily, resulting in a very low sensitivity of WBCT during primary reporting. Motorcycle accidents might predispose for these injuries, and they often cause additional fractures of the extremities. Clinical re-evaluation of patients and secondary re-evaluation of WBCT with preparation of dedicated multiplanar reformations are essential in polytrauma cases to detect osseous injuries of wrist and hand reliably.

**Trial registration:**

The study was registered prospectively on November 17th, 2020, at the German register for clinical trials (DRKS-ID: DRKS00023589).

## Background

Extremity injuries are frequently missed on whole-body computed tomographies (WBCT) depending on the patients‘ability to express pain and the severity of additional injuries. Hence, overlooking of injuries is more likely to occur in cases of old, critically injured, and unconscious patients [1, 2]. The quality of clinical exams and imaging, the experience of physicians, and their interdisciplinary cooperation also influence detection rates of injuries [3]. In an effort to reduce the number of missed injuries, the concept of repeated examinations was introduced: within a defined time-window after an accident, after stabilization of a patient, or after the patient regains consciousness, another thorough clinical exam is conducted, and existing diagnostic imaging is reviewed or complemented [4]. Injuries are then detected with a delay, but fractures of the extremities can usually be treated in a timely fashion [5].

Difficulties during interpretation of WBCT can occur because of artifacts which increase due to unfavorable bedding of patients for WBCT acquisition [6, 7]. Fractures of the carpus are detected on dedicated thin-slice CT of less than 1 mm thickness more accurately [8]. Additionally, reconstruction of each hand and wrist in coronal and sagittal planes after WBCT is intricate which makes thorough examination of these body parts very time-consuming after polytrauma.

Existing literature describes 14–60% extremity fractures among all missed injuries [3, 4, 9]. In a meta-analysis from 2008, 4–33% of extremity fractures were located at the hand or wrist after polytrauma [10]. Recent studies on the prevalence of osseous wrist and hand fractures in polytraumatized patients at specialized centers are often based on patient records or trauma registries [11, 12]. However, imaging studies with modern computed tomography (CT) scanners could possibly visualize more subtle injuries and allow for introduction of a reference test through review by experienced musculoskeletal radiologists. Münn et al. reported 15.5% hand and forearm fractures upon retrospective review of ventilated trauma patients on WBCT [13]. We examined factors influencing the visibility of these injuries on WBCT in this cohort previously [14]. Yet, osseous injuries of the wrist and hand are relevant for all polytrauma patients, especially if long-term damages can be prevented and the working ability can be restored [15, 16].

In 2011 twenty different medical societies first co-published the German S3 guideline for the treatment of polytrauma and the severely injured and defined indications for WBCT after trauma [17]. This current study aims to assess the prevalence, injury patterns, trauma mechanisms, and potentially influencing factors of osseous wrist and hand fractures in a real-life polytrauma patient cohort examined with WBCT at a level 1 trauma center.

## Methods

### Study design

**Fig. 1:**
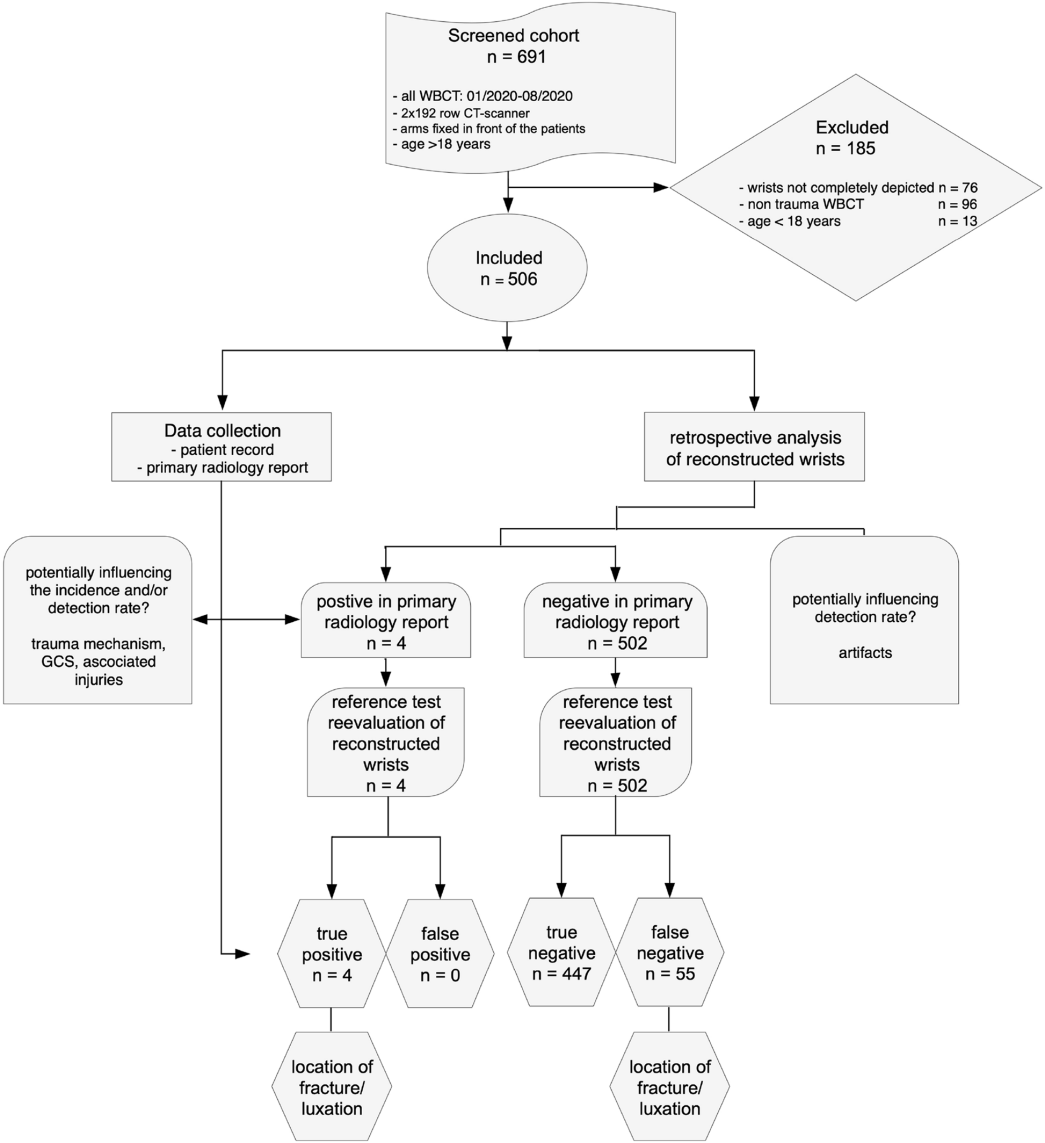
Study design adhering to the STROBE- and STARD-Guidelines [18, 19] with details about enrollment, exclusion, and comparison between index test (primary radiology report on WBCT) and reference test (re-evaluation by musculoskeletal radiologists)

This retrospective analysis was registered prospectively at the German register for clinical trials (DRKS-ID: DRKS00023589) on November 17th, 2020 and conducted in accordance with the Declaration of Helsinki 2013. The institutional review board (Medical Association of Berlin, Germany, Eth-45/20) approved the study protocol and waived the necessity for written consent. The study consisted of seven phases: screening/enrollment, primary radiology report classification, reconstruction of hands/wrists, review by a blinded musculoskeletal radiologist, comparison of the primary report and re-evaluation, search for factors potentially influencing the incidence in imaging and patient records, and calculation of the diagnostic accuracy of WBCT during primary reporting. Figure 1 summarizes the study protocol according to the Strengthening the Reporting of Observational Studies in Epidemiology (STROBE) Initiative [18].

### Screening and enrollment

Screening of consecutive WBCT was conducted from January 2020 to August 2020. Patients were treated due to suspected polytrauma according to the German S3 guideline for the treatment of polytrauma and the severely injured [17]. WBCT was indicated in cases with pathological vital signs, at least two relevantly injured body regions, and/or after relevant trauma mechanisms such as severe road traffic accidents and falls from heights of more than 3 m [20]. Eligible patients had to be ≥18 years of age. Examinations due to a non-emergent cause and studies with incomplete depiction of hands or wrists were excluded from this study.

### Imaging

WBCT were acquired using a double-source CT scanner with two 192 row detectors (Somatom Force, Siemens, Germany). Scans had a slice thickness of 0.75 mm and were performed in spiral technique. The patients‘arms were fixed in front of the patients during imaging.

### Report classification and preparation of cases

Fractures and luxations between the forearm (distal radius and ulnar) and the metacarpal bones were considered positive cases. After inclusion, primary radiology reports (RR) were reviewed manually and classified as either “positive-by-report” or “negative-by-report” by an independent reader. The types and locations of osseous wrist and hand injuries were recorded in a pseudonymized table per patient and side (Excel, Microsoft 365, USA). Coronal and sagittal reconstructions of 1–2 mm thickness were prepared with a post-processing software (IntelliSpace Portal 11, Philips, Netherlands) and stored in a Picture Archiving and Communication System (PACS) (IntelliSpace, Philips, Netherlands) separately for each wrist/hand.

### Reference test and discrepancy review

Reconstructed images were reviewed by two musculoskeletal radiologists blinded to the primary radiology reports. Osseous injuries were recorded for each wrist/hand. Uncertain cases were discussed to determine a conclusive result. Results of the reference test were classified as “positive-by-reference” or “negative-by-reference”. The presence of beam-hardening, motion, or metal artifacts, and artifacts induced by contrast-agent in the veins of the upper extremities were recorded by the reviewers.

Comparison of primary RR and reference test resulted in true positive, false positive, true negative, and false negative cases. False negative cases were further examined by an independent reader for secondary imaging (plain radiographs, CT, or MRI) and delayed diagnosis.

### Patient records

For final data collection, records of included patients were reviewed for age, sex, trauma mechanism, vigilance upon primary presentation, and during hospitalization on the intensive care unit (ICU) using the Glasgow Coma Scale (GCS), additional injuries of the body, as well as the therapy of hand/wrist injuries. Cases of patients with missed hand/wrist injuries which had been discharged without correct diagnosis and therapy were examined by a board-certified hand surgeon for therapeutic consequences. Based on this review, the medical need for contacting these patients was determined.

### Endpoint analysis and statistical analysis

Our reporting adhered to the Standards for Reporting of Diagnostic Accuracy (STARD) statement and recommendations [19]. Results of primary reporting on WBCT and secondary reporting on additional imaging compared with that of the reference standard (review by musculoskeletal radiologists) were reported as true positives (TP), false positives (FP), true negatives (TN), and false negatives (FN). The diagnostic accuracy was expressed as sensitivity, specificity, positive (PPV) and negative (NPV) predicted values with 95% Clopper Pearson [21] confidence intervals (CI).

Associations between the likelihood of suffering osseous wrist and hand injuries and various parameters (localization of osseous wrist/hand injuries, additional injuries, artifacts, trauma mechanism, and GCS) were evaluated via Pearson’s chi squared two-sided test. *P*-values < 0.05 were considered statistically significant.

Descriptive statistics included arithmetic mean, standard deviation (SD), minimum and maximum (range), and absolute (n) and relative (%) proportions. Missing values were not imputed but presented for each variable if existing. The SPSS software package for Windows, version 28 (IBM, Armonk, NY, USA) was employed for all statistical analyses.

## Results

Six hundred ninety-one WBCT were screened according to the inclusion and exclusion criteria between January 2020 and August 2020 at the study center (Fig. 1). Seventy-six cases were excluded because of incomplete depiction of wrists, 96 WBCT were performed for non-traumatic causes, and 13 minors were excluded from the study.

Five hundred six consecutive WBCT of 137 (27.1%) female and 369 (72.9%) male patients were included with a mean age of 53 (18–98) years. Basic demographics were similar in patients with and without osseous injuries of wrist and hand (Table 1).

**Table 1:** Characteristics of polytraumatized patients

Variable	No wrist/hand injuries	Wrist/hand Injuries	Total
n	447	59	506
Mean age, years (SD [range])	53.4 (19.7 [18 to 98])	49.9 (17.2 [18 to 88])	53.0 (19.5 [18 to 98])
Gender, n (%)			
Male	324 (72.5)	45 (76.3)	369 (72.9)
Female	123 (27.5)	14 (23.7)	137 (27.1)
ICU, n (%)			
Yes	147 (32.9)	22 (37.3)	169 (33.4)
No	300 (67.1)	37 (62.7)	337 (66.6)
GCS at ER (SD [range])	12.9 (4.3 [3 to 15])	13.9 (2.9 [3 to 15])	13.0 (4.2 [3 to 15])
GCS at ICU (SD [range])	9.7 (5.9 [3 to 15])	11.6 (5.4 [3 to 15])	9.9 (5.9 [3 to 15])
Motorcycle accident, n (%)*			
Yes	45 (10.1)	13 (22.0)	58 (11.5)
No	398 (89.0)	45 (76.3)	443 (87.5)
Missing	4 (0.9)	1 (1.7)	5 (1.0)
Additional extremity injury, n (%)**			
Yes	165 (36.9)	58 (98.3)	223 (44.1)
No	281 (62.9)	1 (1.7)	282 (55.8)
Missing	1 (0.2)	0	1 (0.1)

*SD* Standard Deviation, *ICU* Intensive Care Unit, *GCS* Glasgow Coma Scale, *ER* Emergency Room, *p = 0.006, **p < 0.001.

### Primary analysis

The radiology reports of WBCT identified *n* = 4 osseous injuries of the wrist and hand. These cases were confirmed during review by the reference as true positive, none of them were false positive cases. Among the remaining 502 cases, the musculoskeletal radiologists identified *n* = 55 additional false negative cases either with fractures (*n* = 42), luxations (*n* = 2), or both fractures and luxations (*n* = 11). Four hundred forty-seven cases were true negative. After review by the gold standard the prevalence of osseous hand and wrist fractures was 11.7% (*n* = 59). 55 (93.2%) of these injuries were missed during primary reporting. Table 2 summarizes the identified cases of fractures and luxations during primary reporting and re-evaluation.

**Table 2:** Cross tabulations of findings during primary reporting versus reference test

		Re-evaluation				Total
		No wrist/hand injury	Fracture	Luxation	Both	
**Primary Reporting on WBCT**	Wrist/hand injury	0	4 (8.7%)	0	0	4 (0.8%)
	No wrist/hand injury	447 (100%)	42 (91.3%)	2 (100%)	11 (100%)	502 (99.2%)
	Total	447	46	2	11	506
**Secondary Imaging and Reporting**	Wrist/hand injury	0	34 (73.9%)	2 (100%)	11 (100%)	47 (9.3%)
	No wrist/hand injury	447 (100%)	12 (26.1%)	0	0	459 (90.7%)
	Total	447	46	2	11	506

The diagnostic accuracy of primary reporting after WBCT for diagnosing osseous wrist and hand injuries was low with a sensitivity of only 6.8% (95% CI 1.9–16.5%) and a specificity of 100.0% (95% CI 99.2–100.0%) (Table 3).

**Table 3:** Measures of diagnostic accuracy of primary reporting on WBCT and secondary reporting after additional imaging

Index test	TP	FP	TN	FN	Sensitivity (95% CI)	Specificity (95% CI)	PPV (95% CI)	NPV (95% CI)
**Primary reporting on WBCT**	4	0	447	55	6.8% (1.9–16.5%)	100.0% (99.2–100.0%)	100.0% (39.8–100.0%)	89.0% (86.0–91.6%)
**Secondary imaging and reporting**	47	0	447	12	79.7% (67.2–89.0%)	100.0% (99.2–100.0%)	100.0% (92.5–100.0%)	97.4% (95.5–98.6%)

*TP* True positive, *FP* False positive, *TN* True negative, *FN* False negative, *PPV* Positive predicted value, *NPV* Negative predicted value, *CI* Confidence interval

After additional, secondary imaging 47 patients with osseous injuries of wrist or hand were identified. Most of these cases (*n* = 36) were identified through additional plain radiographs shortly after WBCT. Secondary imaging using CT (*n* = 6) and MRI (*n* = 1) was less common. 20.3% (*n* = 12) of cases with injuries remained undetected until discharge. The overall diagnostic accuracy after WBCT and secondary imaging for diagnosing osseous wrist and hand injuries in the patient cohort after suspected polytrauma clearly increased when using an additional imaging modality with a sensitivity of 79.7% (95% CI 67.2–89.0%) and a specificity of 100.0% (95% CI 99.2–100.0%) (Table. 2).

### Types of osseous injuries

Ninety-two different osseous injuries were reported, 22 patients suffered multiple injuries. Distal intra-articular radius fractures occurred most frequently (*n* = 24, 26.1%). Fractures of the ulna process (*n* = 18, 19.6%), distal extra-articular radius fractures (*n* = 10, 10.9%), distal ulna fractures (*n* = 6, 6.5%), fractures of Os triquetrum (*n* = 5, 5.4%) and Os pisiforme (*n* = 2, 2.2%), scaphoid fractures (*n* = 4, 4.3%), fractures of hamulus ossis hamati (*n* = 2, 2.2%), and fractures of the various metacarpal bones (*n* = 19, 20.7%) were the other types of fractures described in the collective. Two (2.6%) luxations, one of the distal radio-ulnar joint and one of the radio-joint were observed.

### Association between various parameters and the likelihood of injuries

#### Trauma mechanism

**Fig. 2:**
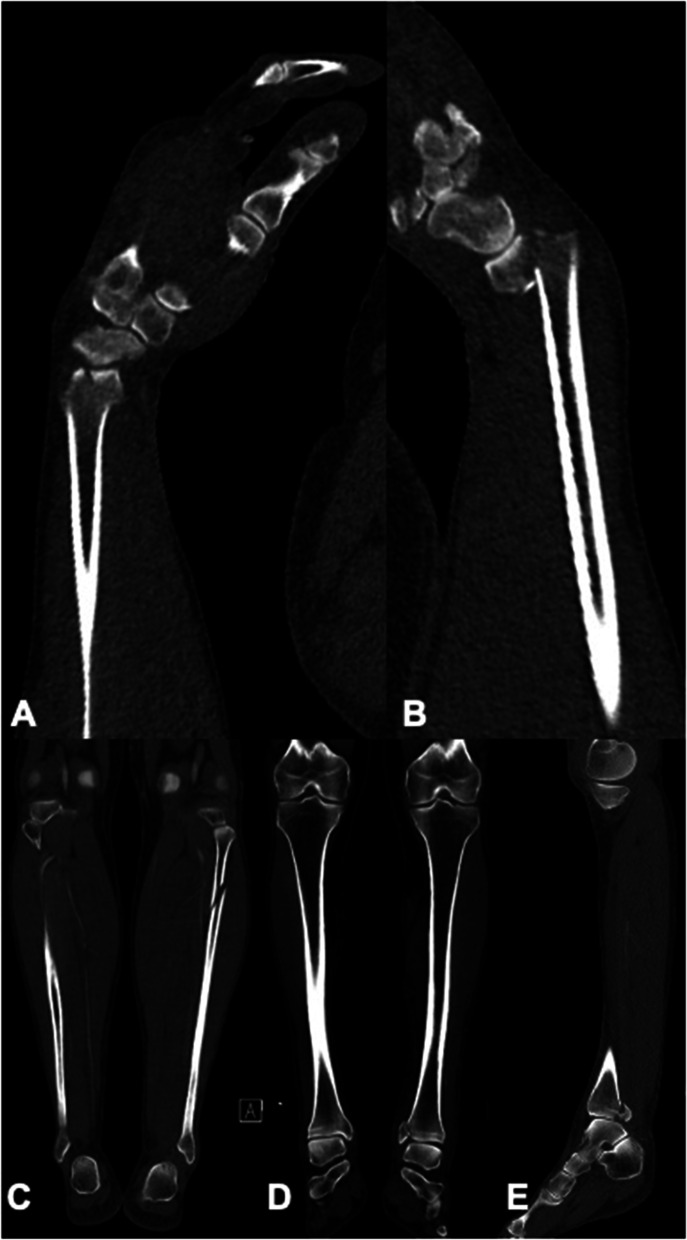
A 52-year-old male patient suffered a motorcycle accident and was examined using WBCT according to the S3 guideline for the treatment of polytrauma and the severely injured [17]. Multiplanar reformations in sagittal planes show distal intraarticular fractures of the radius of both wrists (**A** right side, **B** left side). Imaging showed additional injuries of the left lower extremity with proximal fracture of the fibula on a coronal reformation (**C**), and fracture of the medial malleolus on coronal (**D**) and sagittal (**E**) reformations. Motorcycle accidents might predispose for injuries of wrist and hand, and they often cause additional fractures of the extremities

In the group with osseous injuries, falls accounted for 22 (37.2%), motor-bicycle accidents for 13 (22.0%), car accidents for 10 (16.9%), bicycle accidents for 7 (11.9%), and accidents as pedestrians for 5 (8.5%) of cases. Two accidents were not described in patient records. Motor-bicycle accidents were described significantly less frequent in patients without osseous injuries of wrist and hand (*n* = 45, 10.1%; *p* = 0.006) (Table. 1, Fig. 2). Falls from heights > 3 m were the most common reason for WBCT in this group (*n* = 219, 49.0%).

#### Vigilance

169 (33.4%) patients were treated in the ICU. The fractions of patients treated in the ICU did not differ significantly between patients with and without hand/wrist injuries (37.3% vs. 32.9%, *p* = 0.61). Mean GCS was similar in both patient groups at presentation in the emergency room (ER) and in the ICU. Vigilance was further analyzed for patients with osseous wrist/hand injuries to evaluate the missed rate in a dichotomic fashion for patients with mild impairment but reliable expression, and localization of pain (GCS ≥ 13 points) and patients with more severe cognitive limitations (GCS 3–12 points). The fraction of missed cases did not differ significantly in patients with GCS 3–12 and patients with GCS > 12 (28.6% vs. 19.2%, *p* = 0.56).

#### Injury patterns

58 (98.3%) of patients with osseous injuries of wrist or hand suffered additional injuries of the upper and/or lower extremities while only 165 (36.9%) of patients without wrist/hand injuries showed concomitant extremity fractures (*p* < 0.001), (Fig. 2). The pelvis was injured in 16 (27.1%) patients with wrist/hand injuries compared to 55 patients (12.3%) without wrist/hand injuries (*p* = 0.21). In patients with wrist/hand injuries the head (*n* = 33, 55.9%), thorax (*n* = 18, 30.5%), spine (*n* = 15, 25.4%), and abdomen (*n* = 5, 8.5%) were injured in descending frequency, whereas the head (*n* = 234, 52.3%), thorax (*n* = 162, 36.2%), spine (*n* = 148, 33.1%), and abdomen (*n* = 42, 9.4%) were injured in patients without wrist/hand injuries. More than one organ system was injured in 22 (37.3%) cases with osseous hand and wrist injuries vs. 182 (40.7%) cases without these osseous injuries.

#### Artifacts

Artifacts were reported in 114 (22.9%) cases (*n* = 9/15.3% of cases with and 105/23.5% of cases without osseous wrist or hand injuries). They were mostly caused by contrast agent in distal veins of the hands (*n* = 73, 62.9%) and foreign bodies (*n* = 25, 21.6%). Due to the low percentage of documented artifacts in patients with wrist and hand injuries, the influence of artifacts on primary detection rates on WBCT was not formally tested.

#### Clinical relevance

Twenty-three patients were treated surgically and 24 were treated nonoperatively. Twelve non-displaced fractures of wrist and hand but no luxations remained undetected until discharge: three patients with distal intra-articular radius fractures, three patients with fractures of the 5th metacarpal bone, three patients with fractures of the 2nd metacarpal bone, two patients with fractures of the Os triquetrum, and one patient with a fracture of the distal scaphoid tubercle. A board-certified hand surgeon reviewed the cases and determined that after a time-gap of at least 10 months, physical and radiological follow-up of these patients were no longer medically indicated in almost all cases. All injuries could have been treated nonoperatively with immobilization. Alternatively, the distal intra-articular radius fractures and the fractures of metacarpal bones (*n* = 9) could have been treated surgically. Two patients, one with distal intra-articular radius fracture and one with a fracture of the 2nd metacarpal bone, died due to severe injuries. However, two young patients, one with distal intra-articular radius fracture and one with distal scaphoid tubercle fracture were informed and invited back for follow-up radiographs and examination motivated by the employer’s liability insurance association. Both fractures were consolidated properly without signs of post-traumatic arthrosis.

## Discussion

### Incidence

The frequency of osseous injuries of the wrist and hand on WBCT was 11.7% in this cohort of patients after severe trauma. Distal intra-articular radius fractures occurred most frequently (26.1%). In recent imaging and register studies incidences varied between 15.5% for fractures of hand and forearm in ventilated patients and 36.1% for osseous and soft-tissue injuries [12, 13]. Ferree et al. reported an incidence of 3.5% for fractures and dislocations of the hand with a predisposition for fractures of the metacarpal bones (48%) [11]. Our recent study included 32 fractures of the hand, most of these injuries (59.4%) were also fractures of the metacarpal bones.

It appears reasonable, that the frequency of osseous hand and wrist injuries is influenced by the characteristics of the analyzed patient cohort and that the methods of a CT imaging study differ significantly from a retrospective review of patient records or a register study. One reason for a lower proportion of osseous hand and wrist injuries in the current patient collective might be differences in study design. The selection of the screening cohort was based on the German S3 guideline for the treatment of polytrauma and the severely injured [20] and consisted of a typical, real-life cohort of all trauma patients who were examined via WBCT during the study period. An alternate approach through patient records and data bases, including patients based on the severity of injuries, should increase the pretest probability of the examination results [12]. For the same reason, the incidence of osseous wrist and hand fractures might be higher in a cohort of ventilated patients, which have potentially suffered greater severity of injuries or high-impact trauma.

Additionally, our study and the publication by Fritsche-Oestern et al. did not reveal a significant impact of a low GCS on osseous injuries of the hand or wrist [12]. Also, examination of injury patterns of patients with and without osseous injuries of wrist and hand did not support a mere association of the incidence of osseous wrist/hand injuries and the number and severity of other injuries. An accumulation of motorcyclists and patients with associated additional fractures of the extremities sets focus on the mechanics and force of an accident. Osseous injuries of the wrist and hand can follow selective mono-trauma through a fall from a standing height but can be more complex with multiple fractures in high-impact injuries [22, 23]. An understanding of specific trauma mechanisms and associated injuries can be beneficial in suspecting and diagnosing osseous injuries of the wrist and hand.

### Underreporting

Underreporting occurred in 93.2% of osseous wrist and hand injuries, therefore diagnostic accuracy was low for WBCT during primary reporting. After repeated examination, secondary imaging, and reporting, 20.3% of the injuries remained unreported. In literature, the frequencies of missed wrist/hand fractures differ between 4.1 and 32.9% [10, 12, 14]. Missed injuries have been shown to be more likely in intubated and severely injured patients [24, 25]. Previous data also suggests that on-call duty predisposes for missed fractures and other injuries [14, 26]. Nevertheless, reasons for underreporting were not identified by this current study.

In addition, the subtlety of fractures plays an important role during the diagnostic process [27]. Pfeifer and Pape reviewed that 15–22.3% of all missed injuries were clinically relevant [10]. In the process of this current study, 2 of 12 patients with missed injuries were recalled for re-examination and follow-up imaging as suggested by the employer’s liability insurance association. According to a board-specialized hand surgeon, surgical treatment of these 12 missed injuries was not mandatory. However, it cannot be excluded that failure to detect these injuries and to prescribe immobilization led to worsened clinical outcomes.

In an attempt to reduce the number of missed fractures, authors have suggested improvement of training of clinicians and radiologists, and some promoted the importance of radiographs in diagnosing extremity fractures [27–29]. However, the era of WBCT with the ability to examine patients from head to toe, and the basic radiological principle to apply as little radiation as reasonably achievable (ALARA = as low as reasonably achievable), demands for critical discussion about the possibilities of WBCT and concurrent responsibilities of clinicians and radiologists [30]. The availability of high-quality imaging of wrists and hands on WBCT can be regarded as an opportunity to avoid additional radiation exposure through plain radiographs despite their low contribution to the overall radiation exposure of patients [31]. Admittedly, providing multiplanar reformations of each wrist and hand is a time-consuming process and in cases of polytraumatized patients not the primary concern of clinicians and radiologist. But the importance of multiplanar reformations should be undeniable considering the typical unordered position of hands on WBCT and studies about the additional use of multiplanar reformations i.e., for diagnosing scaphoid fractures [32].

### Algorithm to improve detection of osseous wrist and hand injuries

The concept of re-examination and re-evaluation of existing imaging after polytrauma is routine procedure at the study site. In cases of suspected wrist and hand fractures however, clinical suspicion led to additional, mostly radiographic imaging, and increased detection of osseous wrist and hand injuries from 6.8 to 79.7%.

**Fig. 3:**
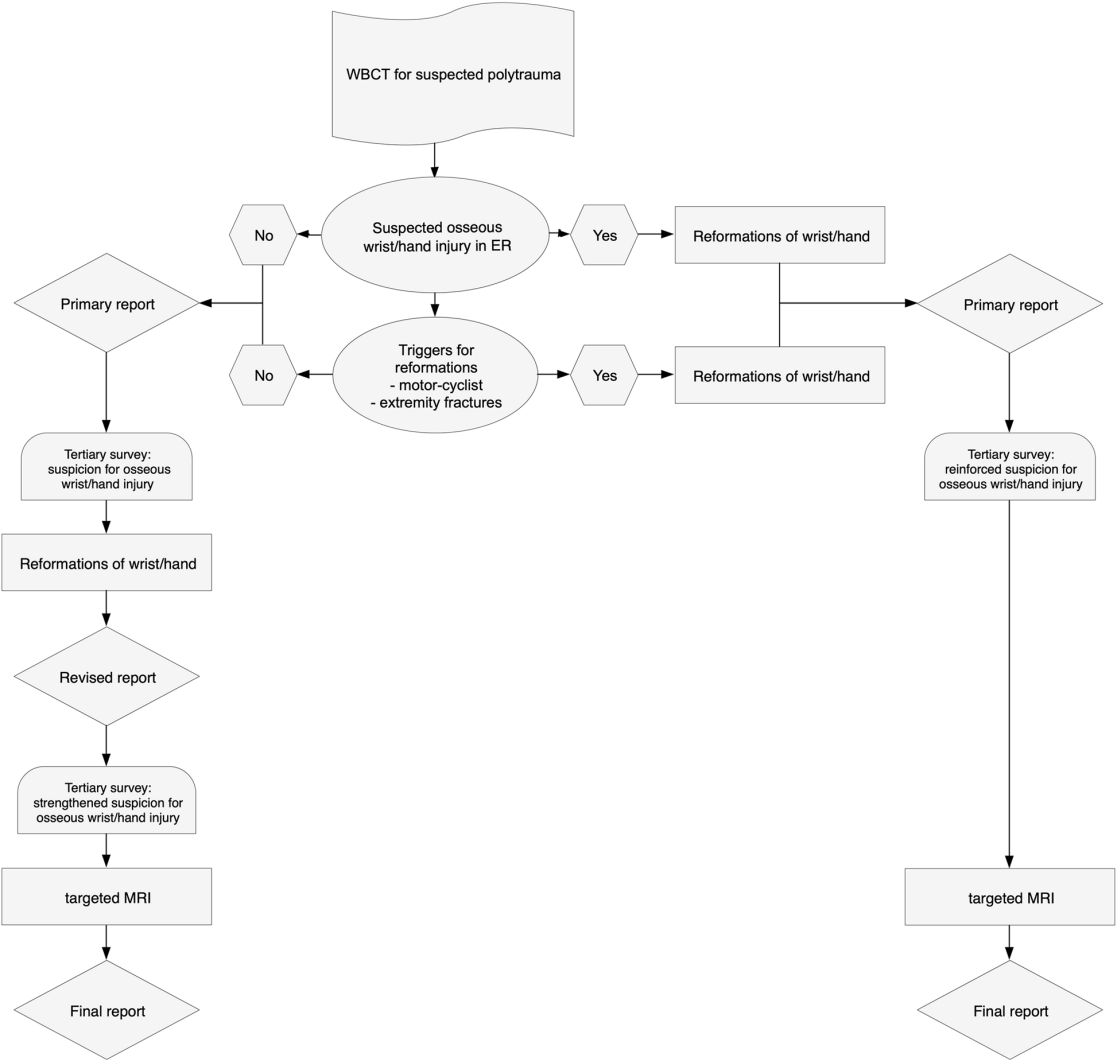
Proposed algorithm for increasing detection of osseous injuries of hand and wrist in accordance with the concept of tertiary survey

In trauma surgery, the concept of tertiary survey is commonly established to ensure re-examination of patients after emergency care, typically within 24 h after admission, and again after patients reach consciousness and are mobilized [33]. Re-evaluation of existing imaging should be part of tertiary surveys and can reduce the number of missed injuries further [34, 35]. Together with primary triggers for profound engagement with a patient’s wrist and hand after polytrauma on WBCT, such as the type of accident and injury patterns, the results of secondary or tertiary clinical examinations and raised suspicions could be regarded as a second chance not only to review existing imaging but to prepare the necessary multiplanar reformations and confirm or exclude osseous injuries without further radiation exposure in a timely fashion [36]. In cases of doubt, targeted MRI might be an option to detect even occult fractures after stabilization of polytrauma patients [37, 38] (Fig. 3). Targeted MRI also enables identification of soft-tissue injuries after (sub-) luxations which reposition spontaneously and are therefore impossible to detect on WBCT reformations. Symptomatic patients with inconclusive CT reformations should thus be examined until all imaging methods are exhausted.

Improvements in intelligent technologies might enable automated multiplanar reformation of complex structures such as hand and wrist from WBCT in the future and further increase detection rates and diagnostic performance [39].

### Limitations

Certain limitations of this study must be addressed. First, the retrospective study design is susceptible to selection bias and missing data. By adhering to the STROBE standards, transparency of the inclusion process of consecutive patients was essential as a countermeasure. Secondly, the exploratory study design and lack of sample size calculations results in limited comparisons. On the other hand, significant differences, such as a higher number of motorcyclists among patients with osseous wrist and hand injuries, are likely to be reliable. In addition, despite the single-center design, the large number of cases with a total of 1012 multiplanar reformatted wrists/hands is one of the strengths of the study. Patient records could be accessed in detail and reasons for missing data were reduced. Lastly, interobserver comparison of the reference tests were not part of the study. Nevertheless, all included cases, wrists and hands were reviewed and thus diagnostic accuracy could be measured on a gold standard as demanded by the STARD Guidelines [19].

## Conclusion

Osseous wrist and hand injuries are present in 11.7% on WBCT after polytrauma. 93.2% of injuries were missed primarily, resulting in a very low sensitivity of WBCT during primary reporting. Motorcycle accidents might predispose for these injuries, and they often cause additional injuries of the upper and/or lower extremities (Fig. 2).

Clinical re-examination of patients and careful re-evaluation of WBCT with preparation of specific multiplanar reformations are essential in polytrauma cases to detect osseous injuries of wrist and hand reliably.

Targeted MRI should be performed to identify occult fractures and soft-tissue injuries in symptomatic patients with inconclusive CT results in the post-acute phase after severe injuries.

## Data Availability

The datasets generated and analyzed during the current study are not publicly available due to privacy restrictions but are available from the corresponding author in an anonymized form on reasonable request.

## Abbreviations

WBCT: Whole-body computed tomographies
CT: Computed tomography
DRKS: German register for clinical trials (Deutsches Register Klinischer Studien)
STROBE: Strengthening the Reporting of Observational Studies in Epidemiology
RR: Radiology report
ICU: Intensive care unit
GCS: Glasgow Coma Scale
STARD: Standards for Reporting of Diagnostic Accuracy
TP: True positives
FP: False positives
TN: True negatives
FN: False negatives
PPV: Positive predicted values
NPV: Negative predicted values
CI: Confidence intervals
SD: Standard deviation
ER: Emergency room
ALARA: As low as reasonably achievable
